# MicroRNA-301b-3p facilitates cell proliferation and migration in colorectal cancer by targeting HOXB1

**DOI:** 10.1080/21655979.2021.1962483

**Published:** 2021-09-07

**Authors:** Jianyong Xiong, Lijuan Zhang, Ren Tang, Zhengming Zhu

**Affiliations:** a Second Abdominal Surgery Department, Jiangxi Cancer Hospital of Nanchang University, Nanchang, Jiangxi, China; b Department of Gastrointestinal Surgery, The Second Affiliated Hospital of Nanchang University, Nanchang, Jiangxi, China; c Department of Medical Record Statistics, Jiangxi Cancer Hospital, Nanchang, Jiangxi, China

**Keywords:** Colorectal cancer (CRC), cancer progression, miR-301b-3p, HOXB1

## Abstract

Previous studies revealed that miR-301b-3p was essential to the onset and development of several cancers, but the implied functions of miR-301b-3p in colorectal cancer (CRC) remained largely unclear. The current study is aimed to exploring the potential roles and possible mechanism of miR-301b-3p in CRC. The abundance of miR-301b-3p and HOXB1 in CRC clinical specimens and cell lines was verified using RT-qPCR. The CCK-8, colony formation, wound healing and transwell assays were adopted to evaluate cell proliferation and migration. The interactivity of miR-301b-3p and homeobox B1 (HOXB1) was identified using bioinformatics analysis and dual-luciferase reporter. The results of RT-qPCR indicated that miR-301b-3p was significantly upregulated in CRC clinical specimens and cell lines. Furthermore, overexpression of miR-301b-3p speeds up CRC cell proliferation and migration. Bioinformatics analysis and dual-luciferase reporter verified that HOXB1 acted as the downstream targeted mRNA. Furthermore, silencing of HOXB1 also obviously accelerated the proliferation and migration ability of CRC cells. miR-301b-3p facilitated cell proliferation and migration in CRC, which was partly reversed by overexpressing HOXB1. In conclusion, our findings demonstrated that miR-301b-3p facilitated CRC cell growth and migration via targeting HOXB1. Our results identified that miR-301b-3p served as a significant oncogene in CRC, which may provide a novel biomarker for diagnosis and therapeutic objective for CRC.

## 1. Introduction

Colorectal cancer (CRC), as one of the most common gastrointestinal cancers, seriously threatens people’s health [1–3]. The prevalence of CRC continues to increase in recent these years, especially in people before age 50 years [4,5]. Many reasons may attributed to environmental risk factors, including bad living habits such as high sugar and fat diet, obesity, lack of exercise, drinking, and smoking [4]. Approximately 25% of CRC patients were identified to exist in distant metastases, which were also the leading cause of cancer-related death [4,6]. Currently, the main therapy for CRC is curative resection combined with adjuvant chemotherapy and radiotherapy [7,8]. Regardless of the continuous improvement in treatment for CRC, the patients’ 5-year survival is not satisfactory enough [9,10]. The common diagnostic markers for colorectal cancer include KRAS/NRAS, BRAF, PI3KCA, HER-2, CEA and MSI-H. However, most of the markers are suitable for a variety of tumors, which is not unique to colorectal cancer. Considering that the pathological pathogenesis of CRC remains largely unclear, it is essential to deeply explore its tumorigenesis mechanism, which contributes to providing novel diagnostic markers and molecular targets for CRC.

Some researchers evaluated clinical variables as predictors of colorectal cancer development and progression and explored lncRNA or circRNA as a diagnostic indicator of the occurrence and development of CRC [11–13]. MicroRNAs (miRNAs) are a class of non-coding RNAs containing approximately 20 nucleotides. Increasing studies pointed out that numerous miRNAs were ectopically expressed in CRC tissues and participated in the development and progression of CRC [14]. Yang and colleagues discovered that miR-1301 has significantly low expression in CRC tissues and suppressed the progression of CRC via signal transducer and activator of transcription 3(STAT3) pathway [15,16]. Sun and coworkers revealed that miR-302a inhibited CRC metastasis and cetuximab resistance through nuclear factor I B(NFIB) and CD44^16^. Also, Liu *et al*. demonstrated that miR-140-3p attenuated CRC progression and liver metastasis via BCL9 transcription coactivator (BCL9) and BCL2 apoptosis regulator (BCL2) [17]. Previous studies identified that miR-301b-3p was associated with the development of several carcinomas, such as breast cancer [18], lung adenocarcinoma [19], gastric cancer [20], and hepatocellular carcinoma [21]. However, the potential functions and possible mechanisms of miR-301b-3p in CRC were largely unclear.

Homeobox B1 (HOXB1) is a member of HOX family, which is essential to morphogenesis in all multicellular organisms. Increasing studies have found that HOXB1 participated in regulating nuclear factor kappa B subunit 1(NF-kB) pathway [22], cancer cell proliferation and apoptosis [23]. Also, accumulating studies showed that HOXB1 acted as a significant tumor suppression gene in many malignant tumors including osteosarcoma [22], glioma [24], and lung cancer [23]. Nevertheless, there were no studies exploring the relationship between HOXB1 and CRC.

In this study, we try to find out miRNA as a diagnostic biomarker of colorectal cancer and explain its mechanism. It showed that miR-301b-3p was obviously upregulated in CRC tissues and cell lines. In addition, miR-301b-3p overexpression accelerated the proliferation and migration ability of CRC cells. Furthermore, bioinformatics and luciferase reporter identified that HOXB1 served as the targeted transcript of miR-301b-3p, while si-HOXB1 has the same effect as miR-301b-3p. Subsequent rescue experiments demonstrated that miR-301b-3p boosted proliferation and migration of CRC cells via aiming at HOXB1.

## 2. Materials and methods

### 2.1 Clinical specimens from CRC

Our Institutional Ethics Review Committee approved this study. All study patients had prior written informed consent. Surgical resection of CRC and their adjacent tissues was obtained from 30 patients with positive CRC who endured surgical resection in our hospital from March to October 2020. None of the CRC patients received radiotherapy, preoperative chemotherapy, or other special treatment. All experimental specimens were quickly frozen and stored in liquid nitrogen for later study.

### 2.2 Cell preparation

CRC cell lines Caco-2 (MSI-H), SW480 (MSS), HCT-116 (MSI-H), HT-29 (MSS), DLD-1 (MSI-H) and perennial colon epithelial cell NCM460 were fostered in Dulbecco Modified Eagle's medium (DMEM) containing 10% FBS. The culture environment was 37°C and 5% CO_2_. The cells were divided when met 90% confluence. All cells were detected free of mycoplasma and bacterial contamination. The cells were washed and digested with PBS and 0.25% trypsin, respectively. Then, the cell suspension was centrifuged at 1000 rpm for 5 minutes and seeded into a new culture plate or 6-well plate.

### 2.3 Cell transfection

DLD-1 and HT-29 cells were transfected using miR-301b-3p mimics, HOXB1 siRNA, pcDNA-HOXB1 and corresponding negative controls, respectively. Oligonucleotides and plasmids were synthesized by Aiji Company. Each group contains about 3 × 10^5^ cells. The cells were transfected with Lipofectamine iMAX. The cells (60–70% fusion) were planted in 6-well plates and accomplished with the required oligonucleotides and/or plasmids within 24 hours after adhesion.

### 2.4 RNA extraction and RT-qPCR

The total RNA was distilled by RN001 RNA Quick Purification Kit (Esscience) following the manufacturer’s requirements. The first strand of cDNAs is hifair® Third, the 1st strand cDNA synthesis SuperMax for qPCR (YESEN, Shanghai, China). miR-103b-3p used stem loop-specific primers, HOXB1, and internal reference used traditional oligonucleotide (DT) primer method. The internal reference of HOXB1 was GAPDH, while the internal reference of miR-103b-3p was U6. SYBR was used for fluorescent quantitative PCR. The determination conditions are as follows: (a) pre-denaturation at 95°C within 5 min, (b) denaturation at 95°C for 10 s, (c) annealing at 60°C for 30 s, and (d) extension at 72°C for 30 s and cycle 40 times. The q-RT PCR results were analyzed by 2^–ΔΔCt^ [25]. The primers were as follows: miR-301b-3p RT Stem Primer 5-GTCGTATCCAGTGCGTGT CGTGGAGTCGGCAATTGCACTGGATACGACCTTTGAC-3; miR-301b-3p(F): 5-CAGTGCAATGATATTGTC-3; miR-301b-3p(R): 5-CAGTGCGTGTCGTGGAGT-3; U6(F): 5ʹ-CTCGCTTCGGCAGCACA-3ʹ and U6 (R): 5-AACGCTTCACGAATTTGCGT-3; HOXB1(F): 5ʹ-GGTATGCTCCTGCCGCCTGCA-3ʹ and HOXB1(R): 5ʹ-ATCAGCATAGGCCGGTGCAA-3ʹ; GAPDH(F): CAAGGTCATCCATGACAACTTTG; and GAPDH(R): GTCCACCACCCTGTTGCTGTAG.

### 2.5 CCK-8 assay

CRC cell proliferation was detected by cell counting kit-8 (CCK-8) (Apexbio, MA, USA). At 24 hours after transfection, about 1 × 10^3^ cells/well were seeded in each well of 96-well plate. At 8 hours, CCK8 reagent was added to each well. After incubation for 1 h, the optical cell density was detected in 450 nm microplate reader. After that, the test was repeated every 24 hours until 96 hours.

### 2.6 Colony formation assay

Cells were isolated with 0.25% trypsin and then turbided in DMEM containing 10% FBS after 24 h of transfection. Cell count will be about 2 × 10^2^ cells/holes, and these were incubated on 6-well plate for 10 days. Then, the culture was carried out at 37°C at 5% CO_2_ level. The cells were fixed and stained in 10% formaldehyde and 0.1% crystalline violet dyes for 15 min, washed the cells gently and dried them, and counted clone clusters directly and calculated clone formation rate.

### 2.7 Transwell assay

Transwell experiment was adopted to check the influence of miR-301b-3p on CRC cell migration. HT-29 and DLD-1 cells were planted in the upper chamber, while conditioned medium in the lower chamber. After 20 h, the cells in the upper chamber were stained with 0.05% crystal violet and then observed.

### 2.8 Protein collection and Western blot

The cell protein was extracted with RIPA lysis buffer containing a mixture of protease inhibitors (Invitrogen, Carlsbad, CA, USA). Then, the cells were centrifuged at 4°C at 14,000 rpm for 20 min. Then, all protein concentrations were determined by BCA method. PAGE gel fast preparation kit (PG112) was used to prepare 10% protein gel, and the gel was prepared according to the instructions. After the gel is solidified, the sample lysates after denaturation are loaded into 10% (w/v) Tris-HCl 12 alkyl sulfate polyacrylamide gel electrophoresis (SDS-PAGE) and are set to operate for 1 h under 120 V. The gel was then transferred onto polyvinylidene difluoride (PVDF) film. Then, PVDF was sealed in 5% skim milk for 60 min, and anti-HOXB1 rabbit monoclonal antibody (1:1000, Proteintech, China) and a rabbit monoclonal antibody against GAPDH (1:5000, Proteintech, China) were used. The cells were incubated at 4°C for 18 h. Then, the PVDF films were washed three times in Tris-buffered saline Tween 20 (TBST) and then soaked in rabbit antibody for 30 min at 25°C. Finally, Omni ECL™ Pico light chemistry Kit (SQ202) exposure is used.

### 2.9 Dual-luciferase reporter system

The wild-type (WT) and mutant (mut) HOXB1 vectors were constructed in pmirGLO vector 3`-UTR. The oligonucleotide sequences used for luciferase analysis were as follows: HOXB1-WT 5‘-CCUGGCAUAUUUAUAUUGCACUA-3ʹ; In DLD-1 cell line, 3 ‘UTR-WT or 3ʹ UTR-mut transfection HOXB1-mut 5ʹ-CCUGGCAUAU UUAUAUCATCAGA-3 ‘, miR-NC or miR-mimics, each were 20 nM. Cell luciferase activity was assessed 48 h later by the dual-luciferase reporting system (Promega, Madison, USA). The experiment was carried out according to the method mentioned before [26].

### 2.10 Statistical analysis

SPSS 22.0 (SPSS Software, Chicago, IL, USA) was used for analyzing the results, while GraphPad Prism version 8.0 (GraphPad software, La Jolla, CA, USA) was used for drawing figures. The mean standard deviation was applied for statistical measurement. One-way ANOVA or Student *T*-tests were applied to compare the distinctions in these groups. If *P < 0.05 and **P < 0.01, the difference is considered to be statistically significant.

## 3. Results

Previous studies revealed that miRNAs participated in the regulation of many key steps in tumor development and progression, including the persistence of tumor proliferation signals, the activation of metastasis and invasion, the escape of growth inhibition, and the induction of vascular production. This study aims to find out the existing relationship between miR-301b-3p and HOXB1 and to elucidate their roles in regulating the proliferation and migration of CRC cell lines. In this study, we used CRC cell lines and tissue samples to verify our results. The outcomes of RT-qPCR showed that miR-301b-3p was highly expressed in CRC clinical specimens and cell lines. Overexpression of miR-301b-3p accelerated CRC cell proliferation and migration. Bioinformatics and dual-luciferase reporter assays verified that HOXB1 acted as the downstream targeted mRNA. Furthermore, silencing of HOXB1 also obviously accelerated growth and migration of CRC cells. Rescue experiments revealed that miR-301b-3p facilitated CRC cell progression, which was partly reversed by overexpressing HOXB1.

### 3.1 miR-301b-3p is overexpressed in CRC specimens and cell lines

**Figure 1.**
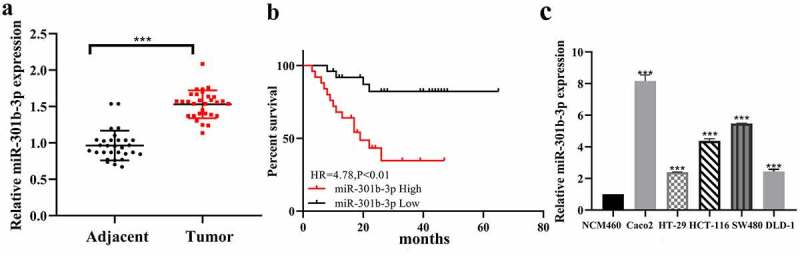
The detection of miR-301b-3p suggested that it is upregulated in colorectal cancer. (a) Expression of miR-301b-3p in CRC tissue. (b) K–M plotter analyze the overall survival of miR-301b-3p. (c) Expression of miR-301b-3p in cell lines. *P< 0.05 and **P < 0.01

We first examined the amount of RNA of miR-301b-3p in CRC and adjacent tissues. RT-qPCR showed that miR-301b-3p expression was higher in CRC tissues than that in adjacent normal tissues (Figure 1 (a)). Also, miR-301b-3p was associated with malignant prognosis in CRC patients (Figure 1 (b)). Consistently, we further verified that miR-301b-3p was obviously upregulated in CRC cell compared to normal colon epithelial cells (Figure 1 (c)). Of these, DLD-1 and HT-29 ranked as the lowest expression level among all the CRC cells, and they were represented as MSI-H(DLD-1) and MSS(HT-29), two different types of CRC, so we chose them to perform subsequent functional experiments.

### 3.2 miR-301b-3p overexpression accelerates proliferation and migration in CRC cells

**Figure 2.**
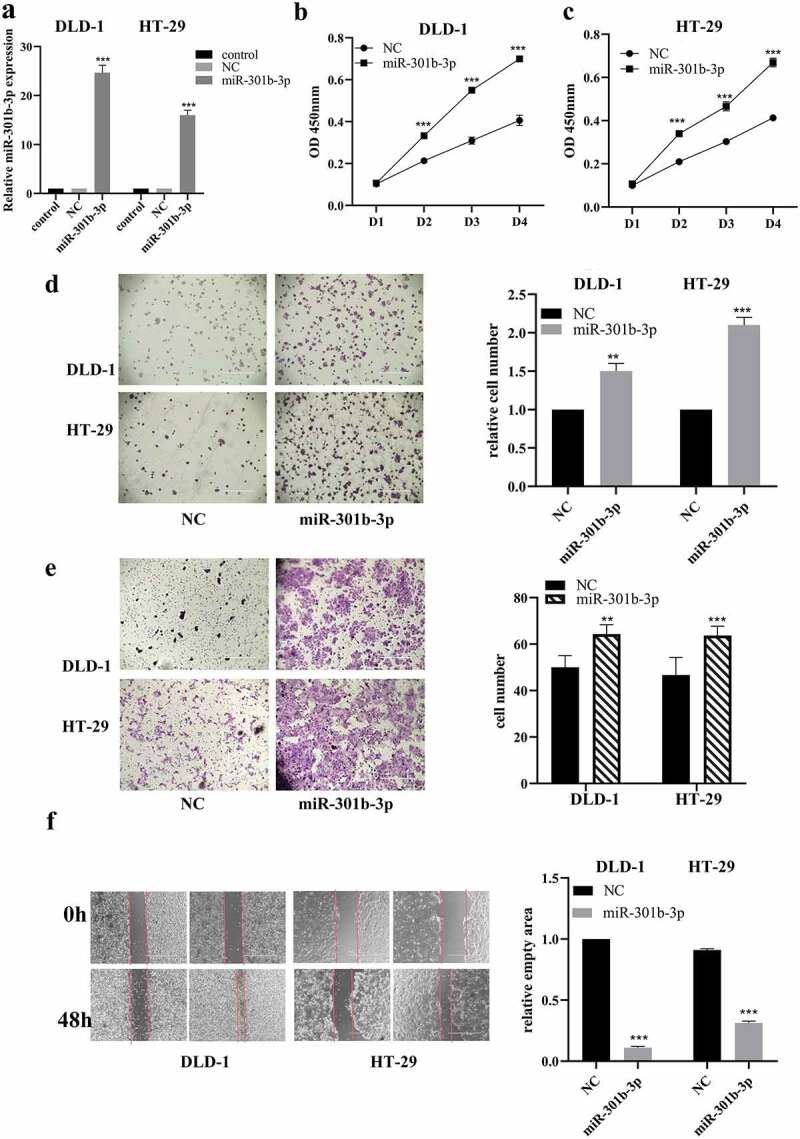
MiR-301b-3p mimics promoted cell growth and invasion in CRC in vitro. (a) Assessment of miR-301b-3p mimics transfection in DLD-1 and HT-29. (b,c) CCK-8 analysis with miR-301b-3p mimics or NC in DLD-1 and HT-29. (d) Colony formation test for cell growth rate with miR-301b-3p mimics in DLD-1 and HT-29. (e,f) Wound-healing and transwell assay for cell migration with miR-301b-3p mimics in DLD-1 and HT-29. *P < 0.05 and **P < 0.01

We tried to investigate the function of miR-301b-3p on proliferation and migration in CRC. RT-qPCR suggested that miR-301b-3p was significantly upregulated in DLD-1 and HT-29 cell lines after transfected with its mimics as exhibited in Figure 2 (a). Moreover, CCK-8 assay and colony formation assay were used to examine the proliferation ability of CRC cells. As shown in Figure 2 (b–d), miR-301b-3p overexpression significantly increased the growth rate of DLD-1 and HT-29 cells. Similarly, using transwell and wound-healing assay, transfection of its mimics significantly promoted cell migration in DLD-1 and HT-29 cells (Figure 2 (e-f)). Collectively, these results hinted that miR-301b-3p can significantly promote cell proliferation and migration in CRC, which suggested that miR-301b-3p may serve as a possible oncogene in CRC.

### 3.3 HOXB1 acts as a target of miR-301b-3p in CRC

**Figure 3.**
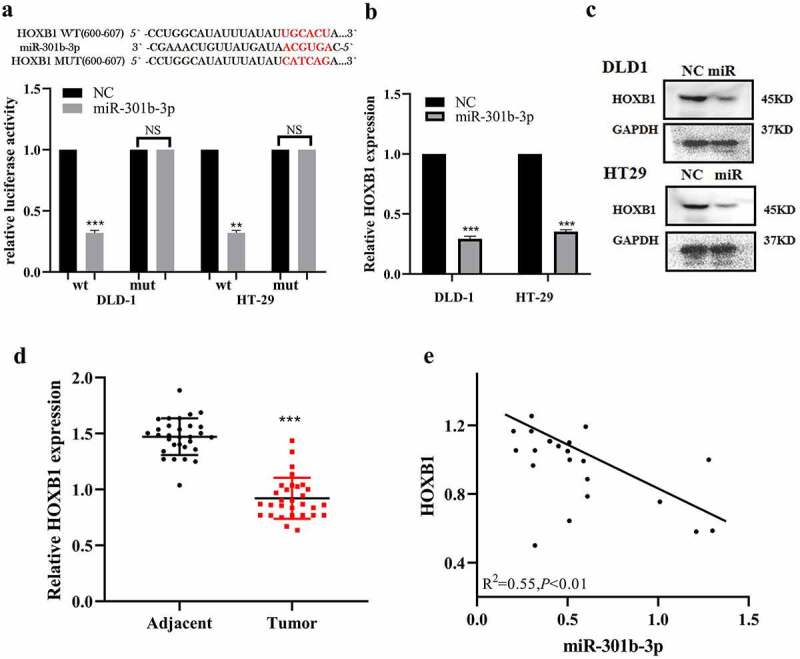
Targeting of HOXB1 by miR-301b-3p in CRC. (a) 3ʹ-UTR region of HOXB1 with the binding site for miR-301b-3p in WT and mut plasmids. The luciferase activity of HOXB1 WT or MUT 3ʹ-UTR with miR-301b-3p mimics in CRC. (b,c) The mRNA and protein level of HOXB1 affected with miR-301b-3p mimics in HT-29 and DLD-1. (d) The expression of HOXB1 mRNA is unregulated in adjacent of colorectal tissues. (e) The relationship of HOXB1 and miR-301b-3p in CRC. (*R*^2^ = −0.55, *P*< 0.01). *P < 0.05 and **P < 0.01

HOXB1 belongs to the HOX family, which plays a vital role in morphogenesis. Increasing studies revealed that HOXB1 served as a significant tumor suppressor in several cancers [22–24]. Interestingly, TargetScan database predicted that the 3ʹ-UTR of the HOXB1 mRNA was complementary to miR-301b-3p, which suggested that HOXB1 could be a putative downstream target of miR-301b-3p (Figure 3(a) upper). In order to verify the binding site between miR-301b-3p and HOXB1, dual-luciferase report experiment was used to detect the change of luciferase activity. Obviously, miR-301b-3p can reduce the luciferase activity in the HOXB1-3`-UTR-WT group, while no significant luciferase activity alteration was identified in HOXB1-3‘-UTR mut group (Figure 3(a) lower). Furthermore, miR-301b-3p overexpression obviously suppressed the abundance of HOXB1 in mRNA and protein level (Figure 3(b–c)). Meanwhile, RT-qPCR also found that the expression level of HOXB1 was less in cancer tissues than that in paracancerous (Figure 3(d)). Pearson correlation analysis revealed that the expression of HOXB1 was negatively correlated with miR-301b-3p in CRC (Figure 3(e)). Taken together, these findings indicated that HOXB1 acted as a spot transcript of miR-301b-3p in CRC.

### 3.4 HOXB1 knockdown accelerates proliferation and migration in CRC

**Figure 4.**
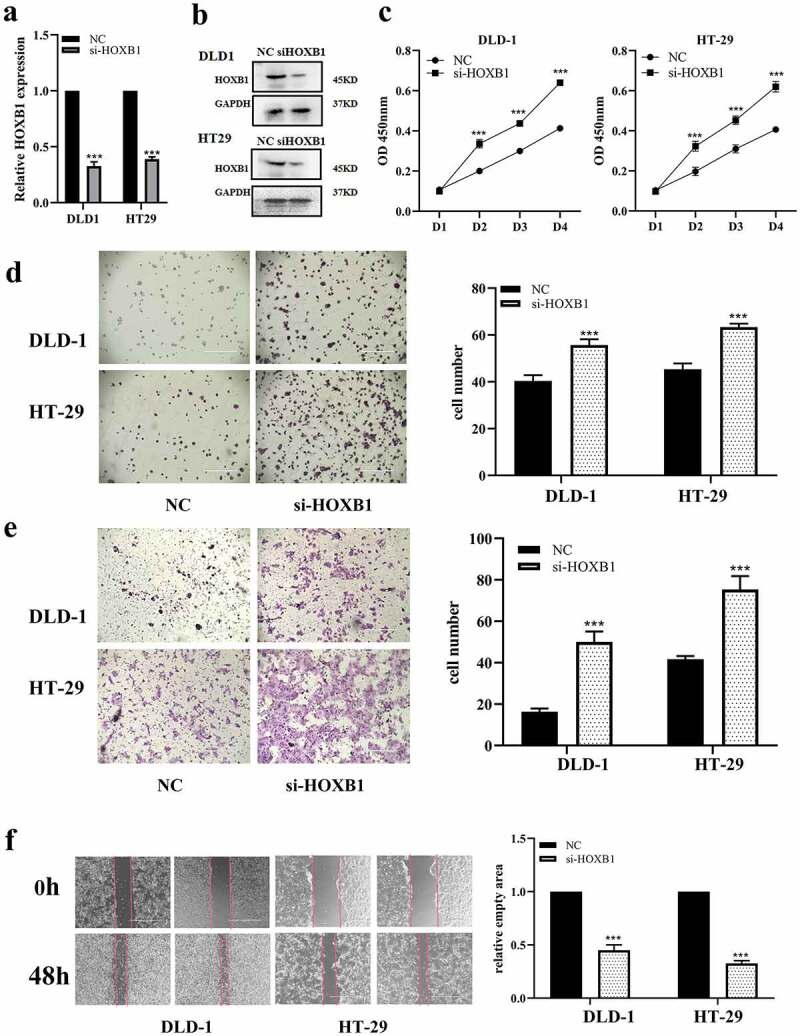
HOXB1 promotes CRC growth and migration while it is knocking down. (a,b) The mRNA and protein of HOXB1 affected by si-HOXB1 siRNAs in CRC. (c) CCK-8 assay with si-HOXB1. (d) Colony formation test for cell growth with si-HOXB1 in CRC. (e,f) Transwell and wound-healing assay for cell migration with si-HOXB1 in CRC. *P < 0.05 and **P < 0.01

Considering that HOXB1 acted as a anti-oncogene in many cancers, we tried to confirm the roles of HOXB1 in CRC. HT-29 and DLD-1 cells were transfected with designed HOXB1 siRNAs [24], and its efficiency was detected using RT-qPCR and Western blot, which showed in Figure 4(a,b), and the expression level of HOXB1 significantly decreased in DLD-1 and HT-29 cells after transfected with si-HOXB1. Furthermore, we further explored the influence of si-HOXB1 on proliferation and invasion of CRC cells. These results of CCK-8 and colony formation assay revealed that the proliferation activities of HT-29 and DLD-1 were significantly increased after transfected with si-HOXB1 (Figure 4(c,d)). Similarly, transwell and wound-healing assay revealed that the migration activity of DLD-1 and HT-29 was increased than that of cells transfected with control after transfected with si-HOXB1 significantly (Figure 4(e,f)). Collectively, our findings suggested that HOXB1 inhibition accelerated proliferation and migration of CRC cells.

### 3.5 miR-301b-3p boosts the progression of CRC via HOXB1

**Figure 5.**
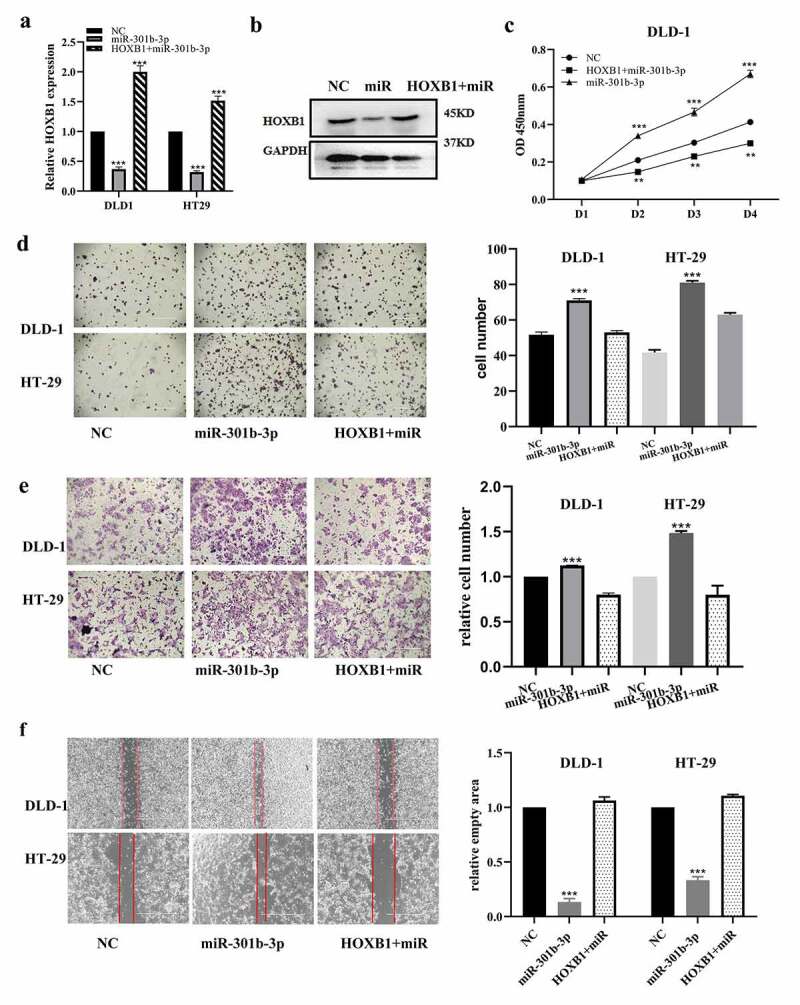
MiR-301b-3p effect rescued by the overexpression of HOXB1 in CRC. (a,b) Expression of mRNA and protein of HOXB1 in CRC under transfection of miR-301b-3p and plasmid of pcDNA-HOXB1. (c) Cell proliferation in CRC under transfection of miR-301b-3p and pcDNA-HOXB1 plasmid. (d) Colony formation analysis for cell growth under transfection of miR-301b-3p and pcDNA-HOXB1 plasmid. (e,f) Transwell and wound-healing test for CRC migration under transfection of miR-301b-3p and pcDNA-HOXB1. *P< 0.05 and **P < 0.01

To ascertain whether miR-301b-3p accelerated the proliferation and migration of HT-29 and DLD-1 cells through HOXB1, we treated them with mimics of miR-301b-3p or NC(mimics) and (or) pcDNA-HOXB1 plasmid. Our results indicated that miR-301b-3p significantly reduced the abundance of HOXB1, which was partly reversed by HOXB1 overexpression (Figure 5(a,b)). Moreover, CCK-8 and colony formation tests showed that miR-301b-3p largely increased the proliferation in HT-29 and DLD-1 cells, which was partly mitigated after transfected with pcDNA-HOXB1 plasmid (Figure 5(c,d)). Consistently, transwell and wound-healing assay revealed that restoration of HOXB1 expression significantly abrogated miR-301b-3p-meditated promoting effect on cell migration of HT-29 and DLD-1 cells (Figure 5(e,f)). To sum up, our findings decipher that miR-301b-3p accelerated the proliferation and migration of CRC cells via targeting HOXB1.

## 4. Discussion

As one of the gastrointestinal cancers with high incidence, pervasiveness of CRC is continuously increasing in China and worldwide. Despite the fact that the treatment for CRC has greatly improved in recent years, the five-year survival rate of CRC is not satisfactory. Therefore, it was essential to seek for new therapeutic approaches. Of these, gene therapy for cancers has been attracting increasing attention recently. At present, we figured out that the miR-301b-3p participated in regulating proliferation and migration of CRC via HOXB1. These results suggested that miR-301b-3p/HOXB1 axis may be a new remedial spot for CRC treatment.

Previous studies identified that miR-301b-3p served as a significant risk factor for several cancers. Fan and colleagues demonstrated that miR-301b-3p was significantly elevated in breast cancer. Furthermore, miR-301b-3p accelerated cell proliferation, migration, and invasion through binding to NR3C2^18^. Also, Liu and coworkers revealed that miR-301b-3p promoted lung adenocarcinoma malignancy through suppressing DLC1 expression [19]. Fan *et al*. demonstrated that miR-301b-3p facilitated the proliferation of gastric cancer cells through attenuating ZBTB4^20^. Similarly, we also verified that miR-301b-3p was obviously overexpressed in CRC spicesman and cell lines in the current study. Furthermore, we demonstrated that overexpression of miR-301b-3p promoted the proliferation and migration of CRC cells. Collectively, our results suggested that miR-301b-3p was a significant contributor to the pathological progress of CRC.

To further clarify its molecular mechanisms, we tried to further seek for the downstream targeted mRNA of miR-301b-3p. Bioinformatics and correlation analysis identified that the expression of HOXB1 was unsympathetically correlated to miR-301b-3p. Previous studies indicated that HOXB1 was a significant tumor suppressor gene in many cancers. Han and coworkers revealed that HOXB1 acted as a tumor suppressor supervised by miR-3175 in glioma [24]. Marina *et al*. found that HOXB1 was significantly downregulated in acute myeloid leukemia. Overexpression of HOXB1 restrained cell proliferation and promoted apoptosis and cell differentiation in the HL60 cell [27]. HOXB1 is also regulated by hsa-let-7 g in inhibiting proliferation of lung cancer cells [23]. Regardless of the important roles of HOXB1 in other cancers, the possible functions of HOXB1 in CRC remained to be unclear. Moreover, we verified that HOXB1 was also downregulated in CRC and HOXB1 knockdown promoted proliferation and invasion in CRC. These findings revealed that HOXB1 was a tumor suppressor in CRC.

To further illuminate the binding relationship between miR-301b-3p and HOXB1, dual-luciferase reporter system confirmed that miR-301b-3p could directly bind to the 3ʹ-UTR of HOXB1 mRNA. Additionally, our results also showed that miR-301b-3p mimics inhibited the expression of HOXB1. Furthermore, we tried to scrutinize whether miR-301b-3p exerted its function via HOXB1. Subsequent rescue experiments involving co-transfected miR-301b-3p mimics and HOXB1 OE plasmid indicated that miR-301b-3p accelerated progression of CRC cells via aiming at HOXB1.

To sum up, our results revealed that miR-301b-3p was significantly upregulated in CRC. miR-301b-3p overexpression promoted proliferation and migration of CRC cells. Furthermore, we identified that HOXB1 served as the targeted gene of miR-301b-3p. Subsequently, rescue experiments identified that miR-301b-3p facilitated the progression of CRC cells via targeting HOXB1. Our study revealed that miR-301b-3p/HOXB1 axis played a critical character in the pathogenesis of CRC, which may contribute to providing novel biomarkers and therapeutic targets for CRC treatment.

## Conclusion

Our findings revealed that miR-301b-3p promoted CRC cell growth and migration through targeting HOXB1. These results suggested that miR-301b-3p served as a oncogene in CRC significantly, which may be a diagnostic biomarker for CRC.

## Research highlight

- MiR-301b-3p is upregulated in colorectal cancer (CRC) and cell lines are accomplished with malignant survival rate.
- Overexpression of miR-301b-3p accelerated proliferation and migration of CRC cells.
- MiR-301b-3p facilitated proliferation and migration in CRC cells via targeting HOXB1.

## Data Availability

All data are fully available without restriction.
